# Prevalence and factors associated with severe pain in the postoperative period of orthopedic surgery

**DOI:** 10.1590/0034-7167-2025-0090

**Published:** 2026-06-12

**Authors:** Juliany Correia de Almeida, Thais Veselich Kamide, Carla Roberta Monteiro Miura

**Affiliations:** I Universidade Federal de São Paulo. São Paulo, São Paulo, Brazil

**Keywords:** Pain, Musculoskeletal Pain, Orthopedics, Postoperative Period, Postoperative Care., Dolor, Dolor Musculoesquelético, Ortopedia, Periodo Posoperatorio, Cuidados Posoperatorios.

## Abstract

**Objectives:**

to determine prevalence and associated factors of severe pain in postoperative orthopedic surgery patients.

**Methods:**

a cross-sectional, quantitative, and analytical study was conducted at a university hospital in São Paulo with 63 postoperative orthopedic surgery patients. Clinical, demographic, and lifestyle variables were collected. The presence of severe pain was assessed using the Numerical Verbal Scale, considering scores ≥7 for severe pain. Statistical analysis employed the Mann-Whitney, Kruskal-Wallis, Fisher’s chi-square, and Logistic Regression tests.

**Results:**

the prevalence of severe pain was 63.5% (95% Confidence Interval: 51.6% to 75.4%). An association was found between mobilization and severe pain. Physical activity was a protective factor against severe pain (*Odds Ratio*=0.3; 95% Confidence Interval=0.1 to 0.8).

**Conclusions:**

the prevalence of severe pain was high and associated with mobilization, with prior physical activity being identified as a protective factor.

## RESUMEN

**Objetivos:**

determinar la prevalencia y factores asociados al dolor intenso en pacientes postoperatorios de cirugía ortopédica.

**Métodos:**

se realizó estudio transversal, cuantitativo y analítico en hospital universitario de São Paulo con 63 pacientes postoperatorios de cirugía ortopédica. Se recopilaron variables clínicas, demográficas y de estilo de vida. La presencia de dolor intenso se evaluó mediante la Escala Verbal Numérica, considerando puntuaciones ≥7 para dolor intenso. El análisis estadístico empleó las pruebas de Mann-Whitney, Kruskal-Wallis, chi-cuadrado de Fisher y Regresión Logística.

**Resultados:**

la prevalencia de dolor intenso fue del 63,5% (Intervalo de Confianza del 95%: 51,6% a 75,4%). Se observó asociación entre la movilización y el dolor intenso. La actividad física fue un factor protector contra el dolor intenso (*Odds Ratio*=0,3; Intervalo de Confianza del 95%: 0,1 a 0,8).

**Conclusiones:**

la prevalencia de dolor intenso fue alta y se asoció con la movilización, identificándose la actividad física previa como factor protector.

## INTRODUCTION

Pain can be defined as “a personal experience that is influenced to varying degrees by biological, psychological, and social factors”, according to the International Association for the Study of Pain criteria^(1)^. Although it is a physiological and universal experience, it has highly subjective characteristics, as its incidence, intensity, and duration vary from person to person. However, it can be stated that surgical patients, especially those undergoing orthopedic surgery, are more susceptible to experiencing intense pain^(2)^.

Scientific findings to date indicate that risk factors for the manifestation of intense pain include age, female sex^(3,4)^, smoking, occupations with frequent physical effort^(3,5)^, high preoperative score on the Verbal Numerical Scale (VNS), previous injury in the surgical region or involvement after a surgical procedure^(6)^, time and type of surgical immobilization^(7)^, anesthetic modality^(3,4,7-9)^, in addition to psychosocial factors such as depression, anxiety, and stress, contributing to pain catastrophizing^(10)^. Furthermore, they also establish a relationship between orthopedic surgery and persistent pain three months after the procedure^(6)^, and greater use of hospital resources, due to longer hospital stay^(3)^.

In addition to the social impacts related to severe pain, there are also personal implications that impact patients’ quality of life, such as functional impairment, increased morbidity, prolonged recovery time, increased duration of opioid use, higher healthcare costs, and a predisposition to developing chronic pain^(11)^.

Despite advances in understanding the mechanisms and risk factors of postoperative pain, there remains a gap in knowledge regarding the prevalence of severe pain and its determinants in patients undergoing orthopedic and traumatic procedures, especially in Brazilian hospital settings. An analysis of existing literature on the subject reveals a lack of evidence that allows for an integrated understanding of how clinical, sociodemographic, and healthcare variables interact and influence the pain experience in these individuals.

In this context, the present study aims to contribute to the advancement of knowledge by identifying the magnitude of the problem and associated factors, providing support for the planning of more targeted and effective nursing interventions in pain management.

## OBJECTIVES

To verify the prevalence and factors associated with severe pain in patients undergoing postoperative orthopedic surgery.

## METHODS

### Ethical aspects

The study was conducted in accordance with national and international ethics guidelines, and was approved by the *Universidade Federal de São Paulo* Research Ethics Committee (Opinion 6,571,665), as provided for in Resolution 466/2012 of the Brazilian National Health Council. Written informed consent was obtained from all individuals involved in the study.

### Study design, period and place

This is a cross-sectional, quantitative, and analytical study, designed and described according to the assumptions recommended by STrengthening the Reporting of OBservational studies in Epidemiology. The study setting was the orthopedics and traumatology ward of a public tertiary hospital in the city of São Paulo.

### Population, sample, inclusion and exclusion criteria

No sample size calculation was performed. All eligible patients were included in the study, which may limit the generalizability of the findings. Adult patients undergoing orthopedic surgery during the current hospitalization, at any stage of the postoperative period, from immediate to late, were included. Patients with cognitive impairment (acute or chronic) or other conditions recorded in the medical records that prevented assessments were excluded.

### Study protocol

For this study, the occurrence of severe pain was considered the dependent variable. The incidence of severe pain episodes was assessed through daily VNS records, recorded by the nursing staff. The VNS is an 11-point verbal pain response scale, ranging from 0 (no pain) to 10 (pain as bad as I can imagine). For this study, severe pain was defined as a reported VNS pain score between 7 and 10^(12)^.

Independent variables were chosen through a bibliographic survey on the subject, and were classified into: demographic and lifestyle variables (age, biological sex, marital status, education, illicit drug use, regular physical activity); clinical variables (presence of comorbidities, history of trauma, Body Mass Index, presence of anxiety symptoms, length of hospital stay, postoperative period, surgical procedure duration, anesthetic modality, and context of intense pain).

To screen for symptoms of anxiety and depression, the Hospital Anxiety and Depression Scale (HADS) was administered. This scale was developed in 1983 by Zigmond and Snaith and later validated by Botega *et al*.^(13)^ in 1995. It consists of 14 items and is subdivided into subscales for anxiety and depression, each with seven items and a score ranging from 0 to 3. Higher scores indicate greater symptoms of anxiety or depression. The total score is 21, with a cut-off point of 8 for each subscale.

Eligible patients were identified through an active search of the ward. After obtaining informed consent from the patient and signing the Informed Consent Form, sociodemographic variables were collected through interviews and applied to the HADS. In addition, records in the electronic medical record were analyzed to identify clinical variables and records of episodes of severe pain. Data collection was conducted from February to August 2024.

### Analysis of results and statistics

The data obtained were entered into the Research Electronic Data Capture platform and analyzed using R version 4.4.0 software. Descriptive analysis was performed on numerical variables (minimum, maximum, mean, standard deviation, and median) and categorical variables (absolute and relative frequency). For numerical variables, their normality was tested using the Shapiro-Wilk test, and none of them presented a normal distribution. Therefore, to compare groups of numerical variables, nonparametric tests (Mann-Whitney and Kruskal-Wallis) were performed. Categorical variables were compared using the chi-square test (if house ≥ 5) or Fisher’s exact test. A logistic regression model was performed using age and length of hospital stay as adjustment variables. The chosen method for variable selection was stepwise (forward selection). A significance level of 5% (p < 0.05) and a 95% Confidence Interval (95%CI) were considered.

## RESULTS

### Sample demographic and clinical characterization

In total, five patients were excluded from the study: three due to cognitive impairment, one due to refusal, and one due to being under 18 years of age. The sample comprised 63 patients, the majority of whom were male (58.7%), with an average age of 52.6 years (minimum of 22 and maximum of 84), single (42.9%), with a high school diploma (46%), and with chronic diseases (77.8%), with arterial hypertension being the most prevalent comorbidity (42.8%), followed by Diabetes Mellitus (23.8%), and 58.7% reported chronic pain.

The most common surgical procedures involved the hip (30.6%), followed by the knee (21%). Most patients had a history of orthopedic trauma (63.5%), with total hip replacement (20.6%) being the most frequently performed surgery. Regarding habits and lifestyle, 61.9% were non-smokers, 65.1% were sedentary, and 96.8% denied using illicit drugs in the last year.

### Prevalence and factors associated with severe pain

The prevalence of severe pain in this sample was 63.5% (95%CI: 51.6% to 75.4%), ranging from one to four episodes.

The highest incidence of these episodes occurred between the first and second postoperative day (40%). The mean VNS score for these episodes was 8.7, with a minimum score of 7 and a maximum of 10.

The operated body regions with the highest incidence of severe postoperative pain were the spine (100%) and calcaneus (100%), followed by the ankle (85.7%), hip (68.4%), femur (66.6%), and knee (61.5%).

Concerning demographic and lifestyle characteristics, it was observed that women (73%), separated/divorced subjects (87.5%), smokers (70.8%), who reported using illicit drugs in the last year (100%) and sedentary individuals (73.1%) were the majority among those who presented episodes of intense pain (Table 1).

**Table 1:** Distribution of patients according to demographic characteristics, lifestyle, and their association with severe pain (N=63), São Paulo, São Paulo, Brazil, 2024

	With intense pain	Without intense pain	*p* value
**Age**			P=0.44a
Minimum	22	26	
Maximum	84	76	
Mean	51.6	54.4	
Median	53	53	
**Sex**			P=0.29b
Female	19 (73.08%)	7 (26.92%)	
Male	21 (56.76%)	16 (43.24%)	
**Marital status**			P=0.12c
Single	20 (74.07%)	7 (25.93%)	
Married	9 (45%)	11 (55%)	
Separated/divorced	7 (87.5%)	1 (12.5%)	
Common-law marriage	1 (50%)	1 (50%)	
Widowed	3 (50%)	3 (50%)	
**Education**			P=0.97c
Completed elementary school	3 (75%)	1 (25%)	
Incomplete elementary school	11 (61.11%)	7 (38.89%)	
Completed high school	18 (64.29%)	10 (35.71%)	
Incomplete high school	3 (75%)	1 (25%)	
Completed higher education	3 (50%)	3 (50%)	
Incomplete higher education	2 (66.67%)	1 (33.33%)	
**Smoking**			P=0.5b
Yes	17 (70.83%)	7 (29.17%)	
No	23 (58.97%)	16 (41.03%)	
**Use of illicit drug**			P=0.53c
Yes	2 (100%)	0	
No	38 (62.3%)	23 (37.7%)	
**Physical activity**			P=0.05b
Yes	10 (45.45%)	12 (54.55%)	
No	30 (73.17%)	11 (26.83%)	

*p values marked with the letter “a” indicate the Mann-Whitney test; p values marked with the letter “b” indicate the chi-square test; p values marked with the letter “c” indicate Fisher’s exact test.*

A higher frequency of intense pain was also observed among those with chronic diseases (69.3%), no history of trauma (70.8%), overweight (Body Mass Index ≥25), and anxiety (68.4%) and depressive symptoms (69.2%) (Table 2).

**Table 2:** Distribution of patients according to clinical characteristics and their association with severe pain (N=63), São Paulo, São Paulo, Brazil, 2024

	With intense pain	Without intense pain	*p* value
**Chronic diseases**			0.13a
Yes	34 (69.39%)	15 (30.61%)	
No	6 (42.86%)	8 (57.14%)	
**Trauma history**			0.50a
Yes	23 (58.97%)	16 (41.03%)	
No	17 (70.83%)	7 (29.17%)	
**Chronic pain**			0.59a
Yes	25 (67.57%)	12 (32.43%)	
No	15 (57.69%)	11(42.31%)	
**Body Mass Index**			0.84b
Minimum	13.45	15.94	
Maximum	36.98	40.82	
Median	26.28	25.92	
Mean	26.53	26.81	
**Postoperative period (days)**			0.71b
Minimum	0	0	
Maximum	15	155	
Mean	3.4	10.26	
**Length of stay**			0.42b
Minimum	2	1	
Maximum	30	193	
Mean	7.25	14.09	
**Hospital Anxiety and Depression Scale**			
**Anxiety**			0.80a
≥ 8	13 (68.42%)	6 (31.58%)	
≤ 8	27 (61.36%)	17 (38.64%)	
**Depression**			0.75c
≥ 8	9 (69.23%)	4 (30.77%)	
≤ 8	31 (62%)	19 (38%)	

*p values marked with the letter “a” indicate the chi-square test; p values marked with the letter “b” indicate the Mann-Whitney test; p values marked with the letter “c” indicate Fisher’s exact test.*

Patients undergoing longer procedures and those who underwent intervention under sedation combined with local anesthesia had a higher frequency of episodes of intense pain (Table 3).

**Table 3:** Distribution of patients according to procedure duration, anesthetic modality and its association with severe pain (N=63), São Paulo, São Paulo, Brazil, 2024

	With intense pain	Without intense pain	*p* value
**Duration of surgical procedure (minutes)**			0.69a
Minimum	60	75	
Maximum	770	500	
Mean	265.05	242.83	
**Anesthetic modality**			0.088b
Sedation + spinal anesthesia	18 (60%)	12 (40%)	
General + spinal anesthesia	7 (87.5%)	1 (12.5%)	
General	5 (41.67%)	7 (58.33%)	
Sedation + local	2 (100%)	0 (0%)	
General + epidural	0 (0%)	1 (100%)	
General + block	5 (83.33%)	1 (16.67%)	
Sedation + block	3 (75%)	1 (25%)	

*p values marked with the letter “a” indicate the Mann-Whitney test; p values marked with the letter “b” indicate Fisher’s exact test.*

None of the demographic, lifestyle, or clinical variables related to the surgical procedure showed a statistically significant association with the occurrence of severe pain.

### Contexts associated with intense pain

As for the context of the intense pain episode, the most frequent were the immediate postoperative period, bath time, during physiotherapy and mobilization, the latter having shown a statistically significant association with episodes of intense pain (p=0.002) (Table 4).

**Table 4:** Context of intense pain (N=63), São Paulo, São Paulo, Brazil, 2024

	With severe pain	Without severe pain	*p* value
**Bath**			0.08a
Yes	6 (100%)	0 (0%)	
No	34 (59.65%)	23 (40.35%)	
**Dressing**			0.08a
Yes	34 (59.65%)	23 (40.35%)	
No	6 (100%)	0 (0%)	
**Walking**			0.11a
Yes	11 (84.62%)	2 (15.38%)	
No	29 (58%)	21 (42%)	
**Physical therapy**			0.29a
Yes	4 (100%)	0 (0%)	
No	36 (61.02%)	23 (38.98%)	
**Mobilization**			0.002a
Yes	13 (100%)	0 (0%)	
No	27 (54%)	23 (46%)	
**Immediate postoperative period**			0.29a
Yes	3 (100%)	0 (0%)	
No	37 (61.67%)	23 (38.33%)	

*p values marked with the letter “a” indicate that Fisher’s exact test was performed.*

In the final Logistic Regression model, age and length of hospital stay (adjustment), mobilization (associated with a higher occurrence of severe pain), and previous physical activity (protective factor) remained. It was found that patients who practice regular physical activity have a 70% lower chance of experiencing severe pain compared to those who do not practice it (Odds Ratio=0.3; 95%CI=0.1 to 0.8; p=0.03).

## DISCUSSION

Concerning the demographic characteristics of the sample, it was observed that the majority of patients in the postoperative period of orthopedic surgeries were male (58.7%), with a mean age of 52.6 years, a history of trauma (61.9%), and had undergone surgical procedures on the lower limbs (77.3%). These findings are similar to those of other studies involving orthopedic patients^(14)^.

Although demographic and clinical variables such as sex, age, comorbidities, and lifestyle habits did not demonstrate a statistically significant association with severe pain, a higher frequency of painful episodes was observed among women, smokers, and sedentary individuals. This finding is similar to that reported in studies that point to these characteristics as potential risk factors for greater pain intensity in the postoperative orthopedic period^(3-5,14)^. The lack of statistical significance may be related to sample size or uncontrolled confounding variables.

A high prevalence of severe pain was identified, with greater recurrence between the first and second day of hospitalization, similar to the findings of another study on the subject, in which the incidence of severe pain after orthopedic surgical procedures was 54%^(3)^, with greater complaints of pain between the first and second postoperative day^(3,7)^. This high prevalence can be explained by both pathophysiological aspects and failures in pain management.

From a pathophysiological perspective, postoperative pain is linked to the hypersensitivity generated by tissue injury, which increases prostaglandin release and induces a reversible increase in synaptic excitability, making any stimulus, even minimal, painful. Furthermore, current pain management methods are often associated with gastrointestinal repercussions, such as nausea and vomiting, adding to patient discomfort^(15)^.

No statistically significant relationship was observed between the presence of depressive or anxious symptoms and the incidence of severe pain itself, although those with depressive and anxious symptoms were the majority among those who experienced severe pain episodes. However, there is scientific support that the presence of these conditions can potentiate the painful event^(16,17)^.

Although smoking was not found to be an associated factor for severe pain in the present study, 70.8% of patients who experienced at least one episode of severe pain were smokers. A study involving 153 postoperative orthopedic surgery patients^(11)^ found that smokers were 2.42 times more likely to experience severe pain.

A significant association was observed between mobilization of the affected limb and severe pain, signaling mobilization as a critical moment for pain exacerbation, especially in lower limb surgeries. This finding highlights the need for specific interventions during the early rehabilitation period, including adequate analgesia and multidisciplinary support, to reduce the impact of pain during movement.

Patients who regularly engage in physical activity were less likely to experience episodes of severe pain. Regular exercise can therefore be considered a protective factor against severe pain, evidence corroborated by other studies demonstrating positive effects of physical activity on the perception of musculoskeletal pain^(18,19)^. This evidence reinforces the need for health policies aimed at promoting physical activity as a component of preparation for elective surgery.

There was no record of the use of non-pharmacological strategies. Therefore, a treatment pattern heavily dependent on a limited number of drugs was observed, as well as a scarcity of other methods. The literature shows that multimodal management of severe pain is the most recommended strategy^(8)^. Multimodality therapy involves the combined use of medications with different mechanisms of action, such as nonsteroidal anti-inflammatory drugs, simple analgesics, and opioid analgesics, to enhance the analgesic effect and reduce the indiscriminate use of opioids. The use of opioids alone is not associated with a lower incidence of pain, but it is linked to a higher incidence of postoperative complications^(20)^.

Non-pharmacological strategies include aromatherapy, music therapy, meditation, guided imagery, massages, hot or cold compresses, and transcutaneous electrical stimulation^(20)^.

It is imperative to highlight that inadequate treatment of severe postoperative pain results in a significant worsening of sleep quality, increased pain perception, decreased early ambulation, increased risk of thrombotic events, altered plasticity of the nervous system and chronification of acute pain, negative physiological repercussions, increased morbidity and mortality, and increased hospitalization period.

The role of nurses in postoperative orthopedic pain management is essential, requiring technical competence, clinical sensitivity and integrated action with the multidisciplinary team to ensure safe, humanized and evidence-based care.

Nurses must systematically and continuously assess pain through the regular use of validated scales, which allow for measuring pain intensity, monitoring its progression, and supporting clinical decision-making. Nurses also work on the safe and timely administration of prescribed analgesics, encouraging the use of multimodal strategies, and implementing complementary interventions. Furthermore, monitoring therapeutic response is an integral part of nursing care, being essential for identifying the effectiveness of interventions and preventing adverse events related to the use of analgesics^(20,21)^.

### Study limitations

In relation to the limitations of this study, it should be noted that the research was conducted in a single center with a small sample size, which may limit the generalizability of results. Furthermore, some data may be lost due to lack of records in electronic medical records, such as systematic recording of pain assessments, impacting the accuracy of the results obtained.

### Contributions to nursing and health

These findings may contribute to new studies on pain in postoperative orthopedic surgery patients. The findings highlight the need for multimodal protocols and nursing staff training to optimize pain control, reduce complications, and accelerate functional recovery.

## CONCLUSIONS

The prevalence of severe pain in the postoperative period of orthopedic surgeries was high, with greater frequency in the first two days after the intervention. An association was found between mobilization and severe pain. Prior physical activity was a protective factor. These findings reinforce the need for systematized strategies for pain management in the postoperative period of orthopedic surgery, with nurses playing a central role in this process.

## Data Availability

The research data are available within the article.
