# Supplementary material for: Prevalence and factors associated with severe pain in the postoperative period of orthopedic surgery
Source: Rev Bras Enferm. 2026 Jun 12;79:e20250090. doi: 10.1590/0034-7167-2025-0090 (PMC13262980; doi:10.1590/0034-7167-2025-0090)
Supplement: Translation

# Translation: Prevalência e fatores associados à dor intensa no pós-operatório de cirurgia ortopédica

**DOI:** 10.1590/0034-7167-2025-0090pt

**Authors:** Juliany Correia de Almeida, Thais Veselich Kamide, Carla Roberta Monteiro Miura

**Affiliations:** I Universidade Federal de São Paulo. São Paulo, São Paulo, Brasil

**Keywords:** Dor, Dor Musculoesquelética, Ortopedia, Período Pós-Operatório, Cuidados Pós-Operatório.

## Abstract

**Objetivos:**

verificar a prevalência e fatores de associados à dor intensa em pacientes em pós-operatório de cirurgias ortopédicas.

**Métodos:**

estudo transversal, quantitativo e analítico, realizado em hospital universitário de São Paulo com 63 pacientes em pós-operatório de cirurgias ortopédicas. Foram levantadas variáveis clínicas, demográficas e de estilo de vida. A presença de dor intensa foi avaliada pelos registros da Escala Verbal Numérica, considerando-se, para dor intensa, escores ≥ 7. Análise estatística empregou os testes Mann-Whitney, Kruskal-Wallis, qui-quadrado de Fisher e Regressão Logística.

**Resultados:**

a prevalência de dor intensa foi de 63,5% (Intervalo de Confiança de 95%: 51,6% a 75,4%). Encontrou-se associação entre mobilização e dor intensa. A prática de atividade física apresentou-se como fator protetor para dor intensa (*Odds Ratio*=0,3; Intervalo de Confiança de 95%=0,1 a 0,8).

**Conclusões:**

a prevalência de dor intensa foi elevada, estando associada à mobilização, sendo a prática prévia de atividade física identificada como fator protetor.

## INTRODUÇÃO

A dor pode ser definida como “uma experiência pessoal que é influenciada, em graus variáveis, por fatores biológicos, psicológicos e sociais”, segundo os critérios da Associação Internacional para o Estudo da Dor^(1)^. Ainda que seja uma experiência fisiológica e universal, ela possui características bastante subjetivas, uma vez que sua incidência, intensidade e duração variam de pessoa para pessoa. No entanto, pode-se afirmar que pacientes cirúrgicos, principalmente aqueles submetidos a cirurgias ortopédicas, estão mais suscetíveis à manifestação de dor intensa^(2)^.

Os achados científicos até o momento indicam que os fatores de risco para manifestação de dor intensa incluem idade, sexo feminino^(3,4)^, tabagismo, ocupações com esforço físico frequente^(3,5)^, alta pontuação pré-operatória na Escala Verbal Numérica (EVN), lesão prévia em região cirúrgica ou acometimento após procedimento cirúrgico^(6)^, tempo e tipo de imobilização cirúrgica^(7)^, modalidade anestésica^(3,4,7-9)^, além de fatores psicossociais como depressão, ansiedade e estresse, contribuindo para a catastrofização da dor^(10)^. Ademais, também estabelecem uma relação entre cirurgia ortopédica e dor persistente três meses após procedimento^(6)^, e maior uso de recursos hospitalares, devido ao maior tempo de internação^(3)^.

Além dos impactos sociais relacionados à dor intensa, há também as implicações pessoais que impactam a qualidade de vida dos pacientes, tais como comprometimento funcional, aumento da morbidade, tempo de recuperação prolongado, ampliação do tempo de uso de opioides, maiores gastos ligados à saúde e predisposição para desenvolvimento de dor crônica^(11)^.

Apesar dos avanços no entendimento sobre os mecanismos e fatores de risco da dor no período pós-operatório, persiste uma lacuna no conhecimento acerca da prevalência da dor intensa e de seus determinantes em pacientes submetidos a procedimentos ortopédicos e traumáticos, especialmente em cenários hospitalares brasileiros. Ao analisar a literatura existente sobre o assunto, nota-se carência de evidências que permitam compreender, de forma integrada, como variáveis clínicas, sociodemográficas e assistenciais interagem e influenciam a experiência dolorosa nesses indivíduos.

Nesse contexto, o presente estudo propõe-se a contribuir para o avanço do conhecimento ao identificar a magnitude do problema e os fatores associados, fornecendo subsídios para o planejamento de intervenções de enfermagem mais direcionadas e efetivas no manejo da dor.

## OBJETIVOS

Verificar a prevalência e fatores associados à dor intensa em pacientes em pós-operatório de cirurgias ortopédicas.

## MÉTODOS

### Aspectos éticos

O estudo foi conduzido conforme as diretrizes de ética nacionais e internacionais, sendo aprovado pelo Comitê de Ética em Pesquisa da Universidade Federal de São Paulo (Parecer n^o^ 6.571.665), como disposto na Resolução nº 466/2012 do Conselho Nacional de Saúde. O Termo de Consentimento Livre e Esclarecido foi obtido de todos os indivíduos envolvidos no estudo por meio escrito.

### Desenho, período e local de estudo

Trata-se de estudo transversal, quantitativo e analítico, cuja elaboração e descrição foram realizadas conforme os pressupostos preconizados pelo *STrengthening the Reporting of OBservational studies in Epidemiology*. O cenário do estudo foi a enfermaria de ortopedia e traumatologia de um hospital público terciário da cidade de São Paulo.

### População, amostra, critérios de inclusão e exclusão

Não foi realizado cálculo amostral. Todos os pacientes elegíveis foram incluídos no estudo, o que pode gerar uma limitação na generalização dos achados. Foram incluídos pacientes adultos submetidos a cirurgias ortopédicas na internação atual, em qualquer fase do período pós-operatório, do imediato ao tardio. Foram excluídos pacientes com incapacidade cognitiva (aguda ou crônica) ou outras condições registradas no prontuário que impediam a realização das avaliações.

### Protocolo do estudo

Para o presente estudo, foi considerada como variável dependente, a ocorrência de dor intensa. A incidência de episódios de dor intensa foi avaliada por meio dos registros diários da EVN, realizados pela equipe de enfermagem. A EVN é uma escala verbal de resposta à dor de 11 pontos, que varia de 0 (sem dor) a 10 (dor tão ruim quanto eu posso imaginar). Para este estudo, a dor intensa foi definida como registro de relato de uma pontuação de dor na EVN entre 7 e 10^(12)^.

A escolha das variáveis independentes se deu por meio de levantamento bibliográfico sobre o assunto, sendo estas classificadas em: variáveis demográficas e de estilo de vida (idade, sexo biológico, estado civil, escolaridade, uso de droga ilícita, prática regular de atividade física); variáveis clínicas (presença de comorbidades, histórico de trauma, Índice de Massa Corporal, presença de sintomas de ansiedade, tempo de internação, período pós-operatório, duração do procedimento cirúrgico, modalidade anestésica e contexto da dor intensa).

A fim de rastrear sintomas de ansiedade e depressão, foi aplicada a *Hospital Anxiety and Depression Scale* (HADS). Trata-se de escala desenvolvida no ano 1983 por Zigmond e Snaith, e posteriormente validada por Botega *et al*.^(13)^ no ano de 1995. É composta por 14 itens, sendo subdividida em subescalas, para ansiedade e depressão, com sete itens cada e pontuação de 0 a 3 para cada item. Quanto maior a pontuação, maiores os sintomas de ansiedade ou depressão. O escore total é de 21 pontos, sendo 8 o ponto de corte em cada subescala.

Os pacientes elegíveis foram identificados por busca ativa na enfermaria. Após a obtenção do consentimento informado do paciente, e mediante assinatura do Termo de Consentimento Livre e Esclarecido, foram levantadas, por meio de entrevistas, as variáveis sociodemográficas, aplicando-se a HADS. Acrescido a isso, procedeu-se à análise de registros no prontuário médico eletrônico, a fim de se levantar as variáveis clínicas e registros de episódios de dor intensa. A coleta de dados compreendeu os meses de fevereiro a agosto de 2024.

### Análise dos resultados e estatística

Os dados obtidos foram inseridos na plataforma de dados *Research Electronic Data Capture*, analisados através do *software* R, versão 4.4.0. Realizou-se a análise descritiva das variáveis numéricas (mínimo, máximo, média, desvio padrão e mediana) e categóricas (frequência absoluta e relativa). Para variáveis numéricas, testou-se sua normalidade através do teste de Shapiro-Wilk, e nenhuma delas apresentou distribuição normal. Portanto, para comparar grupos de variáveis numéricas, foram realizados testes não paramétricos (Mann-Whitney e Kruskal-Wallis). As variáveis categóricas foram comparadas por meio do teste qui-quadrado (se casela ≥ 5) ou teste exato de Fisher. Foi realizado um modelo de Regressão Logística utilizando como variáveis de ajuste idade e tempo de internação. O método escolhido para seleção de variáveis foi o *stepwise* (*forward selection*). Foi considerado nível de significância de 5% (p<0,05) e Intervalo de Confiança de 95% (IC95%).

## RESULTADOS

### Caracterização demográfica e clínica da amostra

Ao todo, foram excluídos da pesquisa cinco pacientes, sendo três por incapacidade cognitiva, um por recusa e um por possuir idade inferior a 18 anos. Compuseram a amostra 63 pacientes, sendo sua maioria do sexo masculino (58,7%), com idade média de 52,6 anos (mínima de 22 e máxima de 84), solteiros (42,9%), com segundo grau completo (46%), portadores de doenças crônicas (77,8%), sendo a hipertensão arterial a comorbidade mais prevalente (42,8%), seguida de Diabetes Mellitus (23,8%), sendo que 58,7% referiram dor crônica.

Os procedimentos cirúrgicos mais frequentes envolviam o quadril (30,6%), seguido do joelho (21%). A maioria dos pacientes possuía histórico de trauma ortopédico (63,5%), sendo a artroplastia total do quadril (20,6%) a cirurgia mais frequentemente realizada. Quanto aos hábitos e estilo de vida, 61,9% eram não tabagistas, 65,1%, sedentários, e 96,8% negaram uso de drogas ilícitas no último ano.

### Prevalência e fatores associados à dor intensa

A prevalência de dor intensa nessa amostra foi de 63,5% (IC95%: 51,6% a 75,4%), variando de um a quatro episódios.

A maior ocorrência desses episódios se deu entre o primeiro e o segundo dia pós-operatório (40%). A média de pontuação à EVN para esses episódios foi de 8,7, com escore mínimo de 7 e, máximo, de 10.

As regiões corpóreas operadas que apresentaram maior incidência de dor intensa pós-operatória foram coluna (100%) e calcâneo (100%), seguidas de tornozelo (85,7%), quadril (68,4%), fêmur (66,6%) e joelho (61,5%).

Quanto às características demográficas e de estilo de vida, observou-se que mulheres (73%), sujeitos de estado civil separado/divorciado (87,5%), tabagistas (70,8%), que relataram uso de droga ilícita no último ano (100%) e sedentários (73,1%) foram maioria entre os que apresentaram de episódios de dor intensa (Tabela 1).

**Tabela 1:** Distribuição dos pacientes quanto às características demográficas, estilo de vida e sua associação com dor intensa (N=63), São Paulo, São Paulo, Brasil, 2024

	Com dor intensa	Sem dor intensa	Valor de *p*
**Idade**			P=0,44a
Mínimo	22	26	
Máximo	84	76	
Média	51,6	54,4	
Mediana	53	53	
**Sexo**			P=0,29b
Feminino	19 (73,08%)	7 (26,92%)	
Masculino	21 (56,76%)	16 (43,24%)	
**Estado civil**			P=0,12c
Solteiro	20 (74,07%)	7 (25,93%)	
Casado	9 (45%)	11 (55%)	
Separado/divorciado	7 (87,5%)	1 (12,5%)	
União estável	1 (50%)	1 (50%)	
Viúvo	3 (50%)	3 (50%)	
**Escolaridade**			P=0,97c
1° grau completo	3 (75%)	1 (25%)	
1° grau incompleto	11 (61,11%)	7 (38,89%)	
2° grau completo	18 (64,29%)	10 (35,71%)	
2° grau incompleto	3 (75%)	1 (25%)	
Ensino superior completo	3 (50%)	3 (50%)	
Ensino superior incompleto	2 (66,67%)	1 (33,33%)	
**Tabagismo**			P=0,5b
Sim	17 (70,83%)	7 (29,17%)	
Não	23 (58,97%)	16 (41,03%)	
**Uso de droga ilícita**			P=0,53c
Sim	2 (100%)	0	
Não	38 (62,3%)	23 (37,7%)	
**Atividade física**			P=0,05b
Sim	10 (45,45%)	12 (54,55%)	
Não	30 (73,17%)	11 (26,83%)	

*Valores de p marcados pela letra “a” indicam realização do teste de Mann-Withney; valores de p marcados pela letra “b” indicam realização do teste qui-quadrado; valores de p marcados pela letra “c” indicam realização do teste exato de Fisher.*

Também se observou maior frequência de dor intensa entre portadores de doenças crônicas (69,3%), sem histórico de trauma (70,8%), com sobrepeso (Índice de Massa Corporal ≥25), e sintomas ansiosos (68,4%) e depressivos (69,2%) (Tabela 2).

**Tabela 2:** Distribuição dos pacientes quanto às características clínicas e sua associação com dor intensa (N=63), São Paulo, São Paulo, Brasil, 2024

	Com dor intensa	Sem dor intensa	Valor de *p*
**Doenças crônicas**			0,13a
Sim	34 (69,39%)	15 (30,61%)	
Não	6 (42,86%)	8 (57,14%)	
**Histórico de trauma**			0,50a
Sim	23 (58,97%)	16 (41,03%)	
Não	17 (70,83%)	7 (29,17%)	
**Dor crônica**			0,59a
Sim	25 (67,57%)	12 (32,43%)	
Não	15 (57,69%)	11(42,31%)	
**Índice de Massa Corporal**			0,84b
Mínimo	13,45	15,94	
Máximo	36,98	40,82	
Mediana	26,28	25,92	
Média	26,53	26,81	
**Período pós-operatório (dias)**			0,71b
Mínimo	0	0	
Máximo	15	155	
Média	3,4	10,26	
**Tempo de internação**			0,42b
Mínimo	2	1	
Máximo	30	193	
Média	7,25	14,09	
***Hospital Anxiety and Depression Scale***			
**Ansiedade**			0,80a
≥ 8	13 (68,42%)	6 (31,58%)	
≤ 8	27 (61,36%)	17 (38,64%)	
**Depressão**			0,75c
≥ 8	9 (69,23%)	4 (30,77%)	
≤ 8	31 (62%)	19 (38%)	

*Valores de p marcados pela letra “a” indicam realização do teste qui-quadrado; valores de p marcados pela letra “b” indicam realização do teste de Mann-Withney; valores de p marcados pela letra “c” indicam realização do teste exato de Fisher.*

Pacientes submetidos a procedimentos com maior tempo de duração e aqueles que passaram por intervenção sob sedação combinada com anestesia local apresentaram maior frequência de episódios de dor intensa (Tabela 3).

**Tabela 3:** Distribuição dos pacientes quanto à duração do procedimento, modalidade anestésica e sua associação com a dor intensa (N=63), São Paulo, São Paulo, Brasil, 2024

	Com dor intensa	Sem dor intensa	Valor de *p*
**Duração do procedimento cirúrgico (minutos)**			0,69a
Mínimo	60	75	
Máximo	770	500	
Média	265,05	242,83	
**Modalidade anestésica**			0,088b
Sedação + raquianestesia	18 (60%)	12 (40%)	
Geral + raquianestesia	7 (87,5%)	1 (12,5%)	
Geral	5 (41,67%)	7 (58,33%)	
Sedação + local	2 (100%)	0 (0%)	
Geral + peridural	0 (0%)	1 (100%)	
Geral + bloqueio	5 (83,33%)	1 (16,67%)	
Sedação + bloqueio	3 (75%)	1 (25%)	

*Valores de p marcados pela letra “a” indicam realização do teste de Mann-Withney; valores de p marcados pela letra “b” indicam realização do teste exato de Fisher.*

Nenhuma das variáveis demográficas, de estilo de vida ou clínicas relacionadas ao procedimento cirúrgico apresentou associação estatisticamente significativa com a ocorrência de dor intensa.

### Contextos associados à dor intensa

Quanto ao contexto do episódio de dor intensa, os mais frequentes foram o período pós-operatório imediato, o momento do banho, durante fisioterapia e a mobilização, tendo este último apresentado associação estatisticamente significativa com episódios de dor intensa (p=0,002) (Tabela 4).

**Tabela 4:** Contexto da dor intensa (N=63), São Paulo, São Paulo, Brasil, 2024

	Com dor severa	Sem dor severa	Valor de *p*
**Banho**			0,08a
Sim	6 (100%)	0 (0%)	
Não	34 (59,65%)	23 (40,35%)	
**Curativo**			0,08a
Sim	34 (59,65%)	23 (40,35%)	
Não	6 (100%)	0 (0%)	
**Deambulação**			0,11a
Sim	11 (84,62%)	2 (15,38%)	
Não	29 (58%)	21 (42%)	
**Fisioterapia**			0,29a
Sim	4 (100%)	0 (0%)	
Não	36 (61,02%)	23 (38,98%)	
**Mobilização**			0,002a
Sim	13 (100%)	0 (0%)	
Não	27 (54%)	23 (46%)	
**Pós-operatório imediato**			0,29a
Sim	3 (100%)	0 (0%)	
Não	37 (61,67%)	23 (38,33%)	

*Valores de p marcados pela letra “a” indicam realização do teste exato de Fisher.*

No modelo final de Regressão Logística, permaneceram as variáveis idade e tempo de internação (ajuste), mobilização (associada à maior ocorrência de dor intensa), e prática prévia de atividade física (fator protetor). Obteve-se que pacientes que praticam atividade física regular têm 70% menos chance de apresentar dor intensa em comparação com os que não praticam (*Odds Ratio*=0,3; IC95%= 0,1 a 0,8; p=0,03).

## DISCUSSÃO

Em relação às características demográficas da amostra, observou-se que a maioria dos pacientes em pós-operatório de cirurgias ortopédicas era do sexo masculino (58,7%), com idade média de 52,6 anos, histórico de trauma (61,9%) e submetida a procedimentos cirúrgicos nos membros inferiores (77,3%). Esses achados são semelhantes aos de outros estudos envolvendo pacientes ortopédicos^(14)^.

Embora variáveis demográficas e clínicas, como sexo, idade, comorbidades e hábitos de vida, não tenham demonstrado associação estatisticamente significativa com a dor intensa, observou-se maior frequência de episódios dolorosos entre mulheres, tabagistas e sedentários. Esse achado é semelhante ao reportado em estudos que apontam essas características como fatores de risco potenciais para maior intensidade dolorosa no pós-operatório ortopédico^(3-5,14)^. A ausência de significância estatística pode estar relacionada ao tamanho amostral ou a variáveis confundidoras não controladas.

Identificou-se uma alta prevalência de dor intensa, de maior recorrência entre o primeiro e segundo dia de internação, semelhante aos achados encontrados em outro estudo sobre o assunto, no qual a incidência da dor intensa após procedimento cirúrgico ortopédico foi de 54%^(3)^, com maiores queixas de dor entre o primeiro e segundo dia pós-operatório^(3,7)^. Essa elevada prevalência pode ser explicada tanto por aspectos fisiopatológicos quanto por falhas na gestão da dor.

Do ponto de vista fisiopatológico, a presença de dor no pós-operatório está ligada à hipersensibilidade gerada pela lesão tecidual, que aumenta a liberação de prostaglandinas e induz ao aumento reversível da excitabilidade sináptica, tornando qualquer estímulo, ainda que mínimo, doloroso. Ademais, os métodos atuais para controle álgico muitas vezes estão associados a repercussões gastrointestinais, como náuseas e vômitos, somando ao desconforto do paciente^(15)^.

Não foi observada uma relação estatisticamente relevante entre a presença de sintomas depressivos ou ansiosos e a incidência de dor intensa propriamente dita, embora aqueles com sintomas depressivos e ansiosos tenham sido maioria entre os que apresentaram episódios de dor intensa. Entretanto, existe respaldo científico de que a presença dessas condições pode potencializar o evento doloroso^(16,17)^.

Embora, no presente estudo, o tabagismo não tenha se apresentado como fator associado para ocorrência de dor intensa, 70,8% dos pacientes que apresentaram ao menos um episódio de dor intensa eram tabagistas. Pesquisa que envolveu 153 pacientes em pós-operatório de cirurgias ortopédicas^(11)^ apontou que tabagistas teriam 2,42 vezes mais chances de apresentar dor intensa.

Observou-se associação significativa entre mobilização do membro acometido e dor intensa, sinalizando a mobilização como momento crítico para exacerbação da dor, sobretudo em cirurgias de membros inferiores. Esse dado destaca a necessidade de intervenções específicas no período de reabilitação precoce, incluindo analgesia adequada e suporte multiprofissional, para reduzir o impacto da dor durante a movimentação.

Pacientes que praticam atividade física regularmente apresentaram menor probabilidade de episódios de dor intensa. A prática regular de exercícios pode, portanto, ser considerada um fator de proteção contra a dor intensa, evidência que é corroborada por outros estudos, os quais demonstram efeitos positivos da atividade física na percepção da dor musculoesquelética^(18,19)^. Tal evidência reforça a necessidade de políticas de saúde voltadas à promoção da atividade física como componente de preparação para cirurgias eletivas.

Não houve registro de emprego de estratégias não farmacológicas. Diante disso, percebeu-se um padrão de tratamento bastante dependente de um número restrito de fármacos, bem como a escassez de outros métodos. A literatura mostra que o manejo multimodal da dor intensa é a estratégia mais indicada^(8)^. A prática multimodal consiste na utilização conjunta de medicamentos com diferentes mecanismos de ação, como anti-inflamatórios não esteroides, analgésicos simples, analgésicos opiáceos, de maneira a potencializar o efeito analgésico e reduzir o uso indiscriminado de opioides. O uso de medicamentos opiáceos, isoladamente, não está associado à menor incidência de dor, no entanto está vinculado à maior incidência de complicações pós-operatórias^(20)^.

Entre as estratégias não farmacológicas, podem-se citar a aromaterapia, musicoterapia, meditação, imaginação guiada, massagens, compressas frias ou quentes, e estimulação elétrica transcutânea^(20)^.

É imperativo destacar que o tratamento inadequado da dor intensa pós-operatória resulta na piora significativa da qualidade do sono, aumento da percepção de dor, diminuição da deambulação precoce, aumento do risco de eventos trombóticos, alteração da plasticidade do sistema nervoso e cronificação da dor aguda, repercussões fisiológicas negativas, aumento da morbimortalidade, e aumento do período de hospitalização.

O papel do enfermeiro na gestão da dor pós-operatória ortopédica é indispensável, requerendo competência técnica, sensibilidade clínica e atuação integrada com a equipe multiprofissional para assegurar um cuidado seguro, humanizado e baseado em evidências.

O enfermeiro deve atuar na avaliação sistemática e contínua da dor por meio do uso regular de escalas validadas, o que permite mensurar a intensidade da dor, monitorar sua evolução e subsidiar a tomada de decisão clínica. O enfermeiro ainda atua na administração segura e oportuna de analgésicos prescritos, no incentivo ao uso de estratégias multimodais, e na aplicação de intervenções complementares. Além disso, o monitoramento da resposta terapêutica é parte integrante do cuidado de enfermagem, sendo indispensável para identificar a eficácia das intervenções e prevenir eventos adversos relacionados ao uso de medicamentos analgésicos^(20,21)^.

### Limitações do estudo

Sobre as limitações deste estudo, pode-se citar que a pesquisa foi realizada em um centro único com pequeno tamanho amostral, o que pode limitar a generalização dos resultados. Ademais, alguns dados podem ser perdidos por ausência de registros nos prontuários eletrônicos, tais como o registro sistemático da avaliação de dor, impactando a acurácia dos resultados obtidos.

### Contribuições para área da enfermagem e saúde

Os resultados encontrados poderão acrescer a novos estudos no campo da dor em pacientes em pós-operatório de cirurgias ortopédicas. Os resultados apontam para a necessidade de adoção de protocolos multimodais e a capacitação da equipe de enfermagem para otimizar o controle da dor, reduzir complicações e acelerar a recuperação funcional.

## CONCLUSÕES

A prevalência de dor intensa no pós-operatório de cirurgias ortopédicas foi elevada, com maior frequência nos primeiros dois dias após a intervenção. Encontrou-se associação entre mobilização e dor intensa. A prática prévia de atividade física apresentou-se como fator protetor. Esses achados reforçam a necessidade de estratégias sistematizadas para o manejo da dor no pós-operatório ortopédico, tendo o enfermeiro papel central nesse processo.

## Data Availability

Os dados de pesquisa estão disponíveis no corpo do artigo.
